# Terminologia anatomica: evolution but not revolution

**DOI:** 10.1007/s12565-024-00760-y

**Published:** 2024-03-20

**Authors:** Jens Waschke

**Affiliations:** Chair of Vegetative Anatomy, Institute of Anatomy, Faculty of Medicine, LMU Munich, Pettenkoferstraße 11, 80336 Munich, Germany

## Viewpoint

Let me start this brief essay with a short disclaimer: I was the delegate for the IFAA (International Federation of Associations of Anatomists) for several years and served on the board of the Anatomische Gesellschaft (AG) during the time when the second edition of the Terminologia anatomica (TA) 2 from 2019 was approved by the IFAA in 2020 (Terminologia Anatomica Second edition, 2019), which was generated by FIPAT (Federative International Programme on Anatomical Terminology). In this context, I participated in the working group of the AG adapting the Terminologia anatomica to establish the Terminologia Anatomica 2023 of the Anatomische Gesellschaft (TA2023AG). As part of these activities, I followed and witnessed several but certainly not all oral and written discussions on that matter.

In addition, I am an editor of anatomy textbooks and atlases and I am involved in the creation of multiple-choice questions for the first medical state exam in Germany. Therefore, I have been in touch with anatomical terminology, also by means other than teaching medical and dental students, and thus my opinions may be biased. Nevertheless, the thoughts and ideas I would like to express are independent of any function I have had in the past and are no official statement by the AG or other institutions but rather my personal point of view. Since I have attended many discussions on this topic, which often were extremely emotional and even fiery, I try to avoid polemics as much as possible.

When I started with anatomy during my medical studies, I was impressed by the stringency of the anatomical terminology which at that time was the Paris Nomina anatomica (PNA) and was used since 1955. This may explain why all anatomy teachers and all recent textbooks and atlases available at that time used the almost identical set of anatomical terms, which was beyond any doubt. Even when historic atlases were considered, a large proportion of terms was either identical or it was, at least, easy to figure out which structure they were referring to. This is due to the fact that in the first terminology, which was introduced in 1895 as the Basle Nomina antomica (BNA), most terms have been fixed (His 1895). This consistency of terms proved to be very helpful to me since sometimes I found discrepancies in the literature regarding the anatomy of a certain structure and it was a relief that at least the term was telling that the different sources were dealing with the same structure. Of course, some of these terms were more logical or even more sophisticated than others, for instance when expressing the origin and insertion of a certain muscle, which facilitated the recall of a structure whereas others appeared complicated and enigmatic.

In my view, the significance of terminology is to have a systematic and consistent compendium of terms on a certain subject that all people can use to allow unequivocal communication if this is possible at all. Anatomy requires such a systematic and consistent set of terms to allow the description of the structure of the human body by words. This was even more important in ancient times when the techniques to produce and copy anatomical images were not sufficient to provide detail on anatomical structures. Even nowadays, it is not possible to show the complete course of all structures by images, even if these would exist. This may change if virtual models become available including all details necessary.

Thus, for teaching anatomy, for writing anatomy books and labelling anatomical figures and last but not least for the creation of unequivocal, legally impeccable examination questions, a consistent set of anatomical terms is strictly required. Especially for the latter it needs to be stated that jurisdiction does not care whether a term is logic or stringent in itself or compared to others from the same terminology. Rather, if a term is not unequivocal and its reference to an anatomical structure is not clear because different versions can be found in the literature, a question can be refutable. This said it is obvious that different terms used for the same structure can cause major problems and also complicate the development of new examinations. Thus, for all anatomists involved in the generation and execution of written and oral exams, as well as in the development of textbooks and other learning material, an anatomical terminology also needs to be consistent over time. Therefore, the modification of terms should be limited and restricted to the necessary corrections of mistakes or grammatical errors and, to address new scientific or moral insights.

When I started teaching anatomy in 2000, I noticed that the terminology was revised and now Terminologia Anatomica (TA) from 1998 was in use which was established by the FCAT (Federative Commitee on Anatomical Terminology). Some terms had been changed and it was easy to adapt to these changes and stay up-to-date. For some terms or the omission of those such as “Diaphragma urogenitale” which was no longer used in the TA because of anatomical differences between women and men it was even kind of a relief that the terminology in anatomy was evolving to adapt to new insights and values. Although it is sad to note that even these well-considered changes in general have not yet made it into clinical use. All these experiences convinced me that the process of optimizing the anatomical terminology by the authorities would be handled with extreme care. Since one of the strengths of the TA was that it was restrictive in the number of changes compared to the PNA, to me it was impossible to imagine that the anatomical terminology at some time would be the subject of a ‘revolution’.

Therefore, I was surprised that when the anatomical societies were asked to decide on a new terminology called TA2, it became obvious that the FIPAT apparently tried to more thoroughly revise the anatomical terminology to improve it. The biggest difference was that “Regular anatomical terms” (RAT) were introduced and Latin terms were assigned as synonyms. Here, I do not question whether the intentions of the FIPAT were the best or whether the composition of the group of experts working on the terminology was sound and balanced. Rather, for me it is critical that a thorough revision of the anatomical terminology (which in the end would lead to changes in 20–30% of anatomical terms compared to the TA from 1998) in itself is counter-intuitive and against the basic principle behind maintaining a terminology. More specifically, a thorough revision greatly diminishes the usefulness of previous anatomy literature. In addition, for our students who learn anatomy to become physicians, major changes to the terminology in their different learning materials would aggravate their task, which is certainly not easy due to the vast number of structures and terms they need to deal with. This holds true even if the logics behind a revision of the terminology and the rules followed thereby are clear and without any disputation.

Because I expect that many protagonists on both sides arguing for or against the strengths of TA2 compared to the TA from 1998 will outline their arguments in detail, I can refrain from doing so. Only a very few points I would like to raise:

1. As outlined above, the modification of a vast number of more than thousand RAT terms needs a very good justification, which is not given for many terms changed in TA2. This holds true especially for all RAT changed without a stringent general rule behind. In this category, I see changes in names for bones from Os sphenoidale in TA to Os sphenoideum in TA2 or Discus articularis in TA to Discus articulationis in TA2.
2. Some goals of the revision may be conflicting with each other: For instance, using the same sequence of subject, its characterizing adjectives and genitive objects for each term by itself is logical. However, the rational to render all terms unique throughout the body and thus to abandon the separate use of terms for parts of different structures such as “Caput mediale” conflicts with the idea to shorten a term by deleting “muscle” when the name of the muscle expresses its function. The latter would require that worldwide all users of a terminology would be able to conclude on the function of muscles based on Latin and Greek terms, which is not the case. In the end, major modifications of terms are required as shown by two examples only: Musculus extensor carpi radialis longus (TA)-Extensor radialis longus carpi (TA2) Musculus flexor carpi ulnaris, Caput ulnare (TA)-Caput ulnare flexoris ulnaris carpi (TA2). Especially for the second example from TA2, I doubt that most students worldwide would be able to decide that this term refers to the part of a muscle and that both terms refer to the same structure. Moreover, since many students do not understand the meaning of the RAT, the intrinsic consistency in different terms is not helpful for them since they have to learn them by heart anyway. Rather, inconsistencies between different learning materials in print or online for them is much more confusing. Moreover, imagine how a table would look like in textbooks where several muscles of the soft palate would read as Musculus palatoglossus and so on, and then followed by Tensor veli palatini and Levator veli palatini. Many such examples could be added.
3. Inconsistencies between different terminologies such as the TA2 approved in 2020 and the Terminologia Neuroanatomica (TNA) approved in 2019, both of which were established by FIPAT and adopted by the IFAA general assembly, need to be avoided by any means, which is also not given.
4. The number of terms increased significantly which is due in part to adding terms used to subdivide anatomical structures from a certain body area. One example is “Pars superficialis compartimenti posterioris antebrachii”, which makes sense but will never be used in any clinical or anatomical setting.

After these considerations I would like to stress that for most of the history of anatomy, a consistent terminology was missing. I like to refer to Josef Hyrtl who stated in his textbook that all attempts to modernize the anatomical terminology had been futile (Josef Hyrtl 1846) and the old terms could be used especially since even the most incorrect terms had not been given up until then. However, in the same way as the content of Hyrtl’s book is impressively timeless, most of the terms he used are still found in TA. Nevertheless, since the first terminology was introduced in 1895 as the Basle Nomina antomica (BNA), today most anatomists agree that a terminology is absolutely necessary. In my opinion, TA should be developed further with care and only where required existing terms should be modified. This would allow constant evolution and adaptation to new scientific insights and moral aspects under the selection of changing circumstances. In addition, constructive alignment is required with other terminologies such as TNA to avoid inconsistencies. In contrast, major changes as shown with the implementation of RATs in TA2 can cause a lot of harm as all revolutions do, and should therefore be avoided.

Based on this, it is understandable why several major anatomical societies decided not to use TA2 in its existing form. Rather it was proposed to combine TA (1998) with Terminologia Neuroanatomica (2017) as a backbone for future anatomical terminology and to introduce new terms as synonyms or to exchange terms as proposed in TA2 only when necessary for good reasons. The second aspect also is important. Anatomy, especially as an old, traditional scientific discipline cannot afford to completely close up and refuse to implement corrections required on the basis of new insight. Thus, is not acceptable to use TA (1998) without any changes for the future. Doing so, anatomy would lose any authority and credibility and could not be considered equal to other scientific or clinical disciplines where constant innovation is required. Also, we could not expect that clinicians would adopt any of the corrections in anatomical terms we may implement. In fact, several of the corrections made by TA2, which fall under the following categories, are a major advantage compared to TA (1998) and should be implemented:

1. Terms where topography was not reflected properly such as Arteria interventricularis inferior (TA2) instead of Ramus interventricularis posterior (TA) related to the fact that Facies inferior cordis was introduced in TA2 as the primary term whereas both Facies diaphragmatica and Facies inferior were used in TA. These new terms should be added as synonyms.
2. Terms changed because scientific insight was provided that a term is incomplete or misleading as for example in Arteria anorectalis superior (TA2) instead of Arteria rectalis superior (TA). These new terms should be added as synonyms also. However, whether or not clinicians will adopt any of these new terms remains to be shown.
3. Several terms need to be replaced as implemented in TA2 when old terms used in TA are inappropriate such as “pudere”-related terms for the female and male genitalia (Zdilla 2022).
4. Also, the fact that TA2 is available online and can be used by everyone without any restrictions is a major effort in the evolution of anatomical terminology.

According to these considerations outlined above, the AG had finalized a new Terminologia anatomica 2023 of the Anatomische Gesellschaft (TA2023AG) by combining TA (1998) with Terminologia Neuroanatomica (2017)a as a backbone and approved it at the general meeting in September 2023. This terminology can now be used for teaching, creating textbooks and atlases and also for the generation of new exam questions. Whenever necessary, it can be adapted in a process for constant evolution since it is laid out to be an ongoing project.

Finally, I hope that these ideas will be taken as constructive criticism with the goal that, in the future, a TA may be developed that can be used and agreed on by all anatomists and all anatomical societies worldwide.

## Data Availability

Not applicable.

## References

[CR1] His W (1895) Die anatomische Nomenclatur. Nomina anatomica, Verzeichniss der von der anatomischen Gesellschaft auf ihrer IX. Versammlung in Basel angenommenen Namen. Leipzig, Veit

[CR2] Hyrtl J (1846) Lehrbuch der Anatomie des Menschen. Verlag von Friedrich Ehrlich, Prag

[CR3] Terminologia Anatomica (1998) International anatomical terminology. FCAT, Thieme, Stuttgart

[CR4] Terminologia Anatomica Second Edition (2019) International anatomical terminology. FIPAT, https://fipat.library.dal.ca/TA2

[CR5] Terminologia Neuroanatomica (2017) International Neuroanatomical terminology. FIPAT, https://fipat.library.dal.ca/tna

[CR6] Zdilla MJ (2022) What is a vulva? Anat Sci Int 97:323–346 35704265 10.1007/s12565-022-00674-7

